# The role of miR-106p-5p in cervical cancer: from expression to molecular mechanism

**DOI:** 10.1038/s41420-018-0096-8

**Published:** 2018-09-20

**Authors:** Yuexiong Yi, Yanyan Liu, Wanrong Wu, Kejia Wu, Wei Zhang

**Affiliations:** Department of Obstetrics and Gynecology, Zhongnan Hospital of Wuhan University, Wuhan, 430071 Hubei People’s Republic of China

**Keywords:** Oncogenes, Oncogenes

## Abstract

This study aims to investigate the role of miR-106b-5p in cervical cancer by performing a comprehensive analysis on its expression and identifying its putative molecular targets and pathways based on The Cancer Genome Atlas (TCGA) dataset, Gene Expression Omnibus (GEO) dataset, and literature review. Significant upregulation of miR-106b-5p in cervical cancer is confirmed by meta-analysis with the data from TCGA, GEO, and literature. Moreover, the expression of miR-106b-5p is significantly correlated with the number of metastatic lymph nodes. Our bioinformatics analyses show that miR-106b could promote cervical cancer progression by modulating the expression of GSK3B, VEGFA, and PTK2 genes. Importantly, these three genes play a crucial role in PI3K-Akt signaling, focal adhesion, and cancer. Both the expression of miR-106b-5p and key genes are upregulated in cervical cancer. Several explanations could be implemented for this upregulation. However, the specific mechanism needs to be investigated further.

## Introduction

At present, with an increase in morbidity and mortality, cancer has become the leading cause of death and a significant public health problem. There are over 500,000 novel cases and approximately 274,000 deaths estimated for cervical cancer (CC) each year all over the world^1^. In 2015, the number of new cases and deaths in China was 4,292,000 and 2,814,000, respectively^2^. CC is the fourth most common cancer in women in the world, and incidence and mortality are still rising^3^. Although the relationship between persistent high-risk human papillomavirus (HPV) infection and CC has been confirmed^4^, the specific molecular cellular network mechanism is still unclear.

Genetic mutations lead to cancer by affecting gene expression and protein function in the cells. However, the dysregulation of microRNA (miRNA) expression is detected in a variety of tumors and is considered to be a significant contributor to the development of cancer in recent years^5,6^. miRNAs are small single-stranded non-coding RNAs that specifically silence gene expression and alter cell or organism phenotypes. Previous studies have confirmed that miRNAs participate in proliferation, apoptosis, morphogenesis^7^, antiviral defense^8^, and tumorigenesis^9^.

Recently, growing evidence reveals that miR-106b-5p plays a critical role in various cancers. Huang and Hu^10^ showed that the upregulation of miR-106b can be observed in the endometrium and knockdown of miR-106b suppresses proliferation and promotes apoptosis. Shi et al.^11^ reported that upregulation of miR-106b-5p exhibited a promoting role in hepatocellular carcinoma (HCC) cell properties and migration, whereas downregulation exhibited an opposite effect. Lu et al.^12^ and Xiang et al.^13^ submitted that overexpression of miR-106b-5p could promote the proliferation and increase the number of metastatic colonies, whereas inhibition would induce cell cycle arrest, suppress cell proliferation, and promote cell apoptosis in renal cell carcinoma.

For CC, miR-106b, the pre-miRNA of miR-106b-5p, also has a pivotal role in occurrence and development. While constructing a miRNA-mRNA network for CC, Ma et al.^14^ found that miR-106b was one of the key nodes in the network. Overexpression of miR-106b promoted the migration of HeLa and SiHa cells significantly while inhibition displayed an opposite phenomenon^15^. However, few studies concern the mechanisms of miR-106b for CC at present. Cheng et al.^15^ found that DAB2 is identified as a direct target of miR-106b and it is inhibited by TGF-β1 partly through miR-106b and is involved in TGF-β1-induced CC cell migration. Piao et al.^16^ reported that miR-106 overexpression and DAB2 knockdown induced epithelial to mesenchymal transition (EMT) of CC cells cultured on substrate. As miR-106b plays an essential role in CC, its molecular mechanisms need to be further studied.

The purpose of this study is to investigate the role of miR-106b-5p in CC by performing comprehensive research on its expression and identify its putative molecular targets and pathways based on The Cancer Genome Atlas (TCGA), Gene Expression Omnibus (GEO), and literature review.

## Results

### Clinical significance of miR-106b-5p

**Fig. 1:**
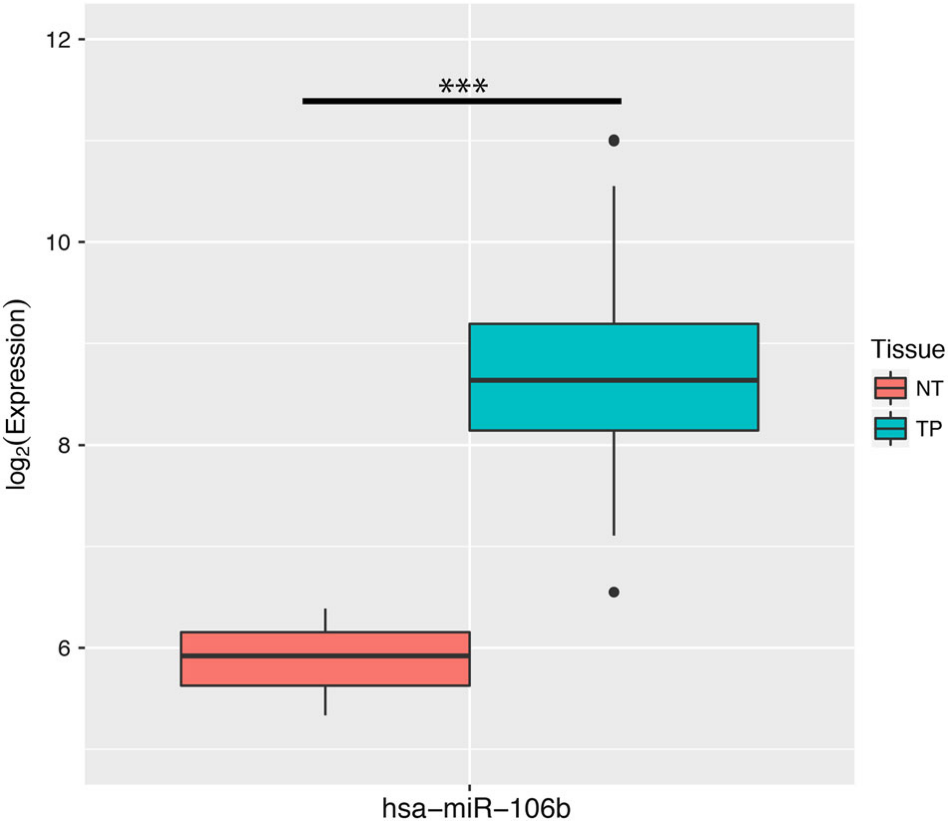
Expression of miR-106b-5p in cervical cancer from TCGA. NT normal tissue, TP primary tumor. There are three samples in the NT group and 309 samples in the TP group. Student's *t*-test is used for the statistical analysis

As the expression data of mature miR-106b-5p are absent in TCGA, a comparison of miR-106b between CC and healthy samples is provided. The expression level of miR-106b is higher in CC (Fig. 1) and significantly associated with the number of metastatic lymph nodes (Cor = 24.510, *P* = 0.006). However, there is no significant correlation in tumor purity, race, pathological M/N/T stage, number of years of birth, histological type, race, radiotherapy, or overall survival (Table 1).

**Table 1:** Correlations between expression of miR-106b-5p and clinical outcomes

Item	Method	Cor.	*P* value	FDR
Number of lymph nodes	Kruskal–Wallis Test	24.510	0.006^a^	0.070
Tumor purity	Spearman Correlation	0.107	0.078	0.430
Race	Kruskal–Wallis Test	7.214	0.125	0.458
Pathology M stage	Wilcox Test	0.025	0.193	0.530
Years to birth	Spearman Correlation	0.063	0.300	0.553
Histological type	Kruskal–Wallis Test	6.047	0.302	0.553
Ethnicity	Wilcox Test	−0.015	0.386	0.606
Radiation therapy	Wilcox Test	0.011	0.545	0.750
Pathology N stage	Wilcox Test	−0.006	0.683	0.758
Pathology T stage	Kruskal–Wallis Test	2.256	0.689	0.758
Overall survival	Cox Regression Test	0.045	0.792	0.792

^a^Significant difference

### Meta-analysis of miR-106b-5p expression

#### Meta-analysis based on TCGA and GEO

**Fig. 2:**
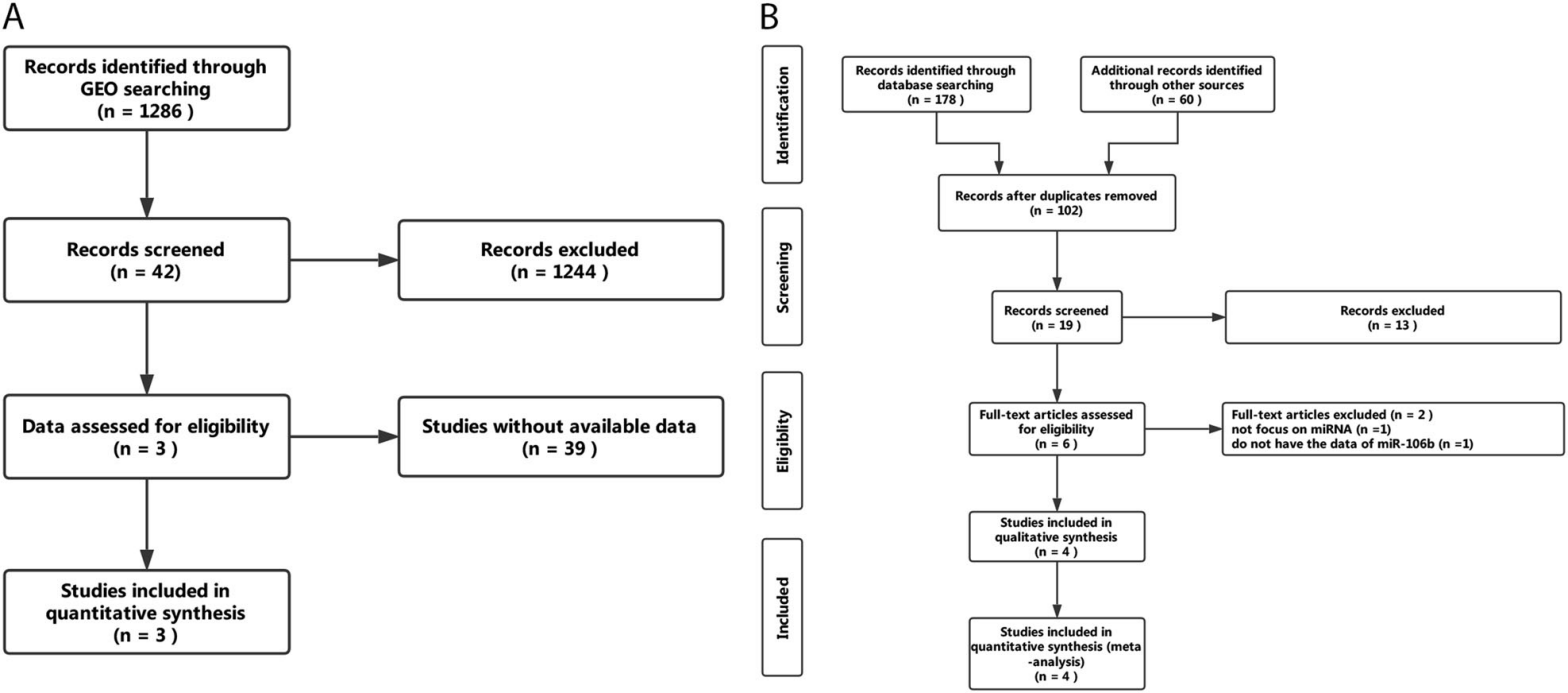
**Searching workflow for the expression of miR-106b-5p between cervical cancer and non-cancerous tissue.** **a** Searching strategy in GEO; **b**Searching strategy in literature review

**Fig. 3:**
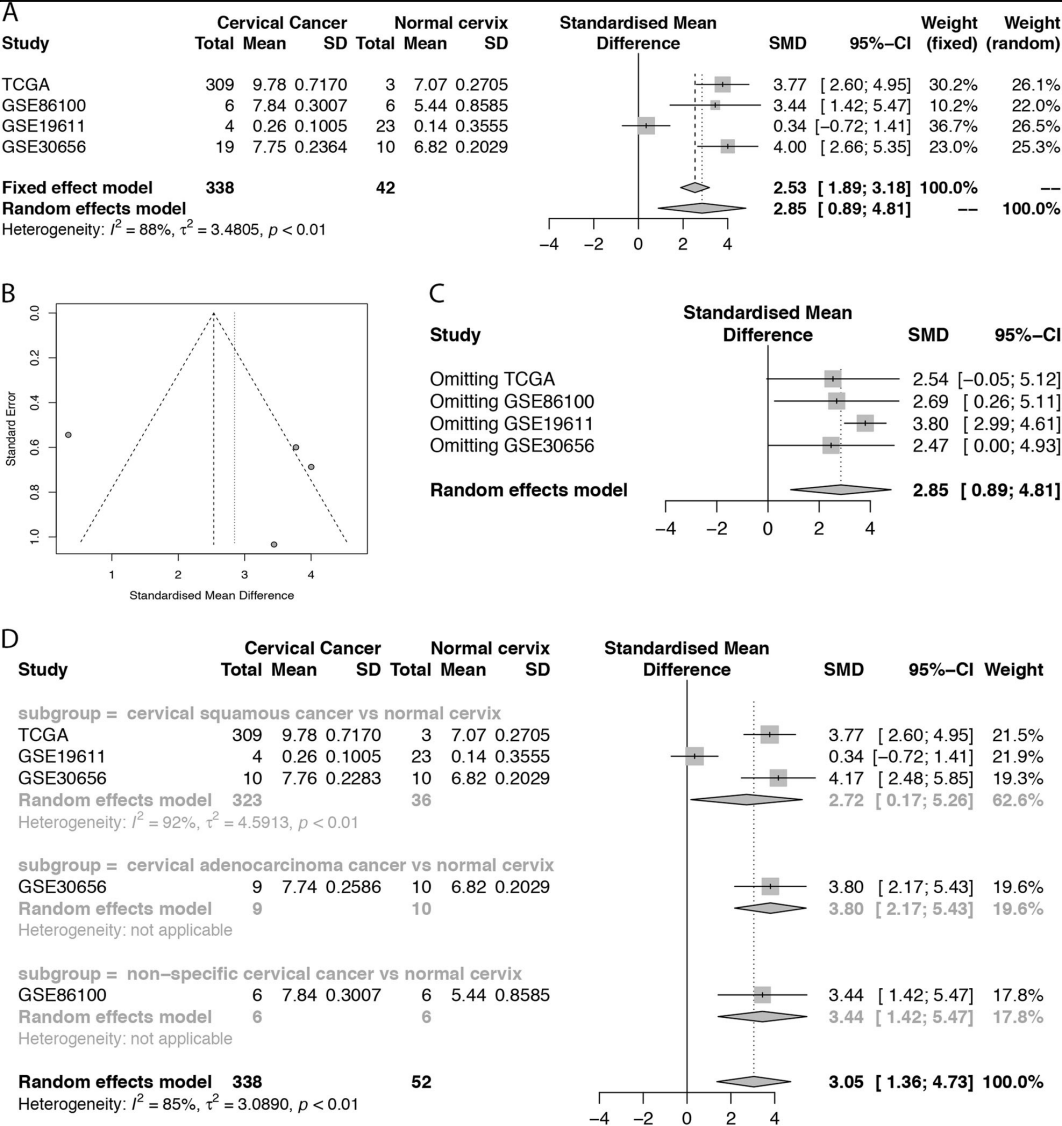
Meta-analysis of miR-106b-5p between healthy and cancerous cervical tissue based on TCGA and GEO. **a** Forest plot of SMD. The expression of miR-106b-5p is significantly higher in cervical cancer tissue; **b** Funnel plot for four studies that are marked as circles. No significant publication bias is detected (*P* = 0.5187); **c** Influence analysis for four studies. No study had an impact on the overall SMD estimation. **d** Subgroup forest plot based on cancer type. As *I*^2^ value is still relatively high, the cancer subtype is not the only source of heterogeneity

A total of 1286 microarrays were obtained from GEO. After careful screening, the three microarrays, GSE86100, GSE19611, and GSE30656, meet the criteria and are included in the analysis (Fig. 2a). The forest plot presents an overall standard mean difference (SMD) of 2.85 (95% confidence interval (CI): 0.89–4.81) with *P* = 0.0045 and *I*^2^ = 88% (random effect used), suggesting that miR-106b-5p is upregulated in CC (Fig. 3a).

A funnel plot of miR-106b-5p expression (Fig. 3b) reveals that no significant publication bias is detected by Egger’s test (*P* = 0.5187). Sensitivity analysis shows that similar results are obtained for the fixed effects models except for a lower difference (SMD: 2.53, 95%CI: 1.89–3.18, *P* < 0.0001).

The influence analysis (Fig. 3c) shows that no study had an impact on the overall SMD estimation because the point estimate for any of the studies is not outside the combined analysis CI and there is no significant statistical change.

Except for a decrease in *I*^2^ values, similar results are obtained in the subgroup analysis of cancer subtypes (Fig. 3d). The results show that the cancer subtype is not the only source of heterogeneity as the *I*^2^ value is still relatively high, but miR-106b-5p continues to be highly expressed in CC tissues.

#### Meta-analysis based on literature review

The workflow for searching is presented in Fig. 2b. Finally, four studies^14,15,17,18^ that met the criteria were selected. Consistent with the result of the meta-analysis, a common pattern of upregulation for miR-106b-5p in CC was reported across the included studies (Table 2).

**Table 2:** Overview of the four studies selected in the literature review

Author	Year	Country	Cancer (*n*)	Normal (*n*)	Result	Detection methods
Cheng et al.	2016	China	19	19	Upregulated	qRT-PCR
Gao et al.	2016	China	30	26	Upregulated	qRT-PCR
Ma et al.	2012	China	8	8	Upregulated	qRT-PCR
Liu et al.	2016	China	10	10	Upregulated	qRT-PCR

### Bioinformatics analyses of miR-106b-5p

#### Screening of candidate genes

**Fig. 4:**
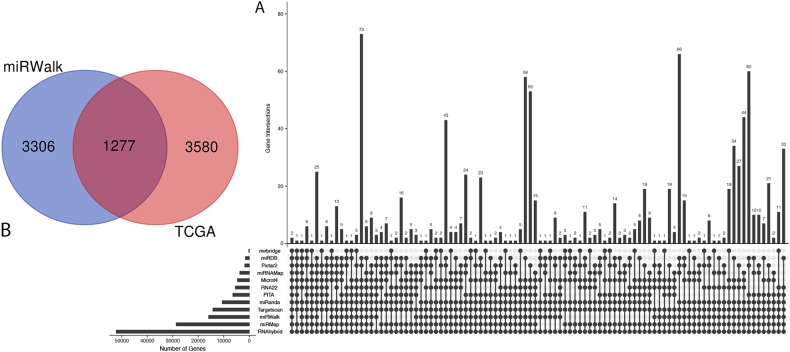
Predication of miR-106b-5p target genes and candidate genes screening. **a** The number of overlapped genes across 12 databases; 10,073 target genes which overlapped at least five databases are obtained. **b** Venn plot for the integration between DEGs and predicted target genes of miR-106b-5p

By analyzing the data from Cervical squamous cell carcinoma and endocervical adenocarcinoma (CESC) with the criterion of log|FC| > 1 and FDR < 0.05, 4857 differentially expressed genes (DEGs) were selected, including 4619 upregulated genes and 238 downregulated genes. Meanwhile, predicting using 12 databases in miRWalk, 10,073 target genes that were overlapping in at least five databases were found (Fig. 4a). After merging DEGs and the predicted target genes, 1277 candidate genes were collected (Fig. 4b).

#### Gene ontology enrichment analysis

**Fig. 5:**
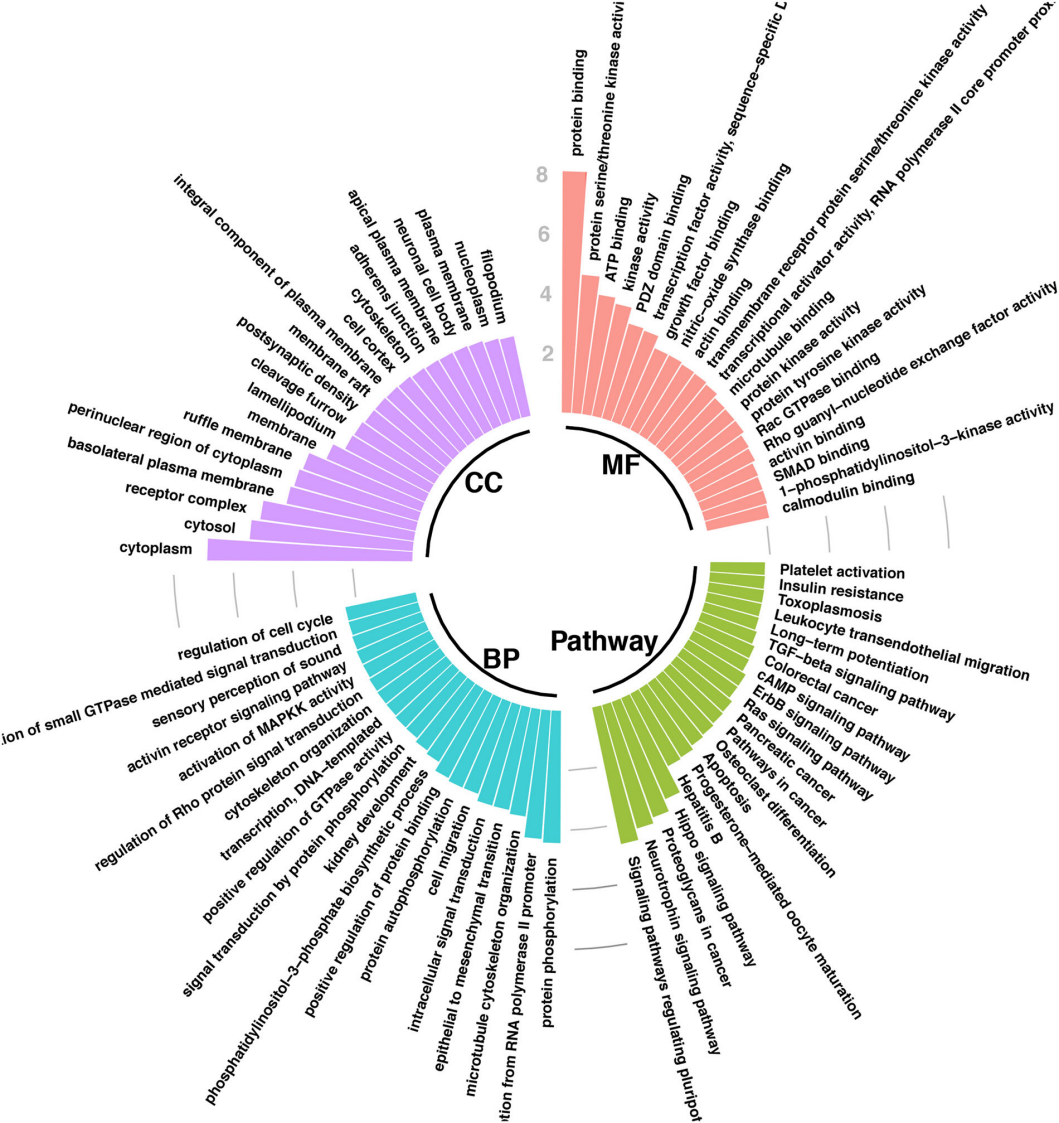
The top 20 items of cellular component (CeC), biological process (BP) pathways, molecular function (MF), and pathways in Gene Ontology (GO) and pathway enrichment analysis for candidate target genes of miR-106b-5p in CC. Values are expressed as −log10 (*P*-value)

The DAVID database was used for Gene Ontology (GO) analysis of the 1277 genes (Fig. 5). Using the criterion of *P* < 0.001, the results showed that while cellular component (CeC), target genes are mainly involved in “cytoplasm”, “cytosol”, “receptor complex”, “basolateral plasma membrane”, “perinuclear region of cytoplasm”, “ruffle membrane”, “membrane”, “lamellipodium”, “cleavage furrow”, “postsynaptic density”, “membrane raft”, “integral component of plasma membrane”, and “cell cortex”. In terms of biological process (BP), the target genes mainly participate in “protein phosphorylation”, “positive regulation of transcription from RNA polymerase II promoter”, “microtubule cytoskeleton organization”, “epithelial to mesenchymal transition”, “intracellular signal transduction”, “cell migration”, “protein autophosphorylation”, and “positive regulation of protein binding”. With regard to MF, these genes are mainly enriched in “protein binding”, “protein serine/threonine kinase activity”, “ATP binding”, “kinase activity”, “PDZ domain binding”, and “transcription factor activity, sequence-specific DNA binding”.

#### Protein–protein interaction network analysis

**Fig. 6:**
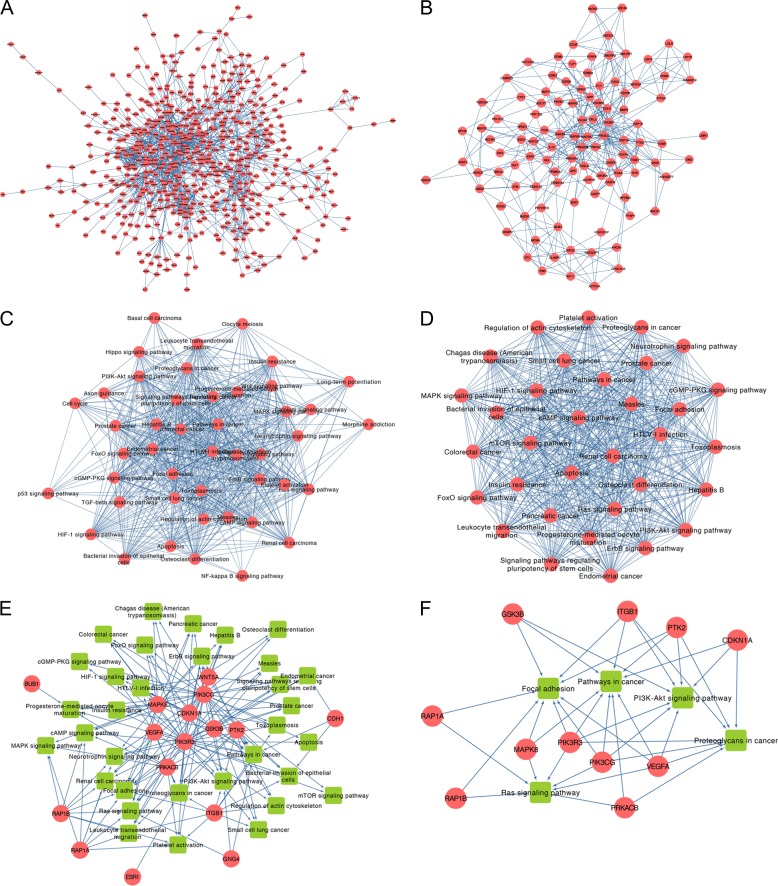
Protein–protein analysis (PPI) analysis of candidate genes and pathway crosstalk and gene-pathway analysis of hub genes. **a** PPI network of candidate genes; **b** Subnetwork of PPI for main nodes extracted according to degree and betweenness being higher than average; **c** Pathway crosstalk analysis of hub genes. The thickness of lines between nodes are represented by the average value of Jaccard coefficient (JC) and overlapping coefficient (OC); **d** Subnetwork of pathway crosstalk extracted by MCODE; **e** Comprehensive gene-pathway network constructed by mapping the hub genes to the subnetwork. The arrow direction between gene and pathway is determined by KEGG. Red circle: genes; green square: pathway. **f** Subnetwork of gene-pathway collected according to the criteria that node’s degree > average

The original network contains 668 nodes and 1779 edges. After cleaning the isolated genes, the main network containing 579 nodes and 1716 edges was obtained (Fig. 6a). By extracting the nodes with degree and betweenness which are higher than average, a subnetwork that contains 110 nodes and 404 edges was gained (Fig. 6b; Table 3).

**Table 3:** Characteristics of main genes

Gene	Degree	Betweenness
Average value	5.928	2064.408
PIK3CG	46	41488.54899
PIK3R3	41	25199.73317
APP	35	32767.23403
MAPK8	33	35071.18269
PRKACB	29	27367.34287
PTK2	28	25355.409
ITGB1	28	17088.50374
SMURF1	27	7546.059618
SMURF2	27	6748.334913
BUB1	26	11066.28571
GNG4	26	10653.80228
H2AFV	25	20305.78635
WNT5A	25	13709.55435
VEGFA	23	20760.79426
KIF23	21	10253.88184
RAD51	21	17892.93805
KIF11	21	5135.989346
CCNF	20	5540.628454
RAP1A	19	7521.72323
PPP2R1B	19	14143.0877
BRCA1	19	6029.273042
NEDD4	19	3192.727805
CLASP1	19	7496.485807
SYK	19	11386.15814
GSK3B	19	18438.62749
DNM2	19	5911.698768
ADCY2	18	7560.286406
ATM	18	9420.324388
CDKN1A	18	18240.66944
RHOT1	18	16492.35297
HIP1R	17	3517.329081
RAP1B	17	5215.72323
MCM4	17	5808.281935
UBE4A	17	2304
PARK2	17	2366.125584
CDH1	17	14376.3563
UBE2I	17	10854.31473
SYNJ2	17	5273.61775
IL10	16	11158.43666
LRP2	16	3590.998058
KIAA0319	15	3475.143506
RACGAP1	15	5861.489126
ACTR1A	15	3744.522591
PRDM10	15	14008.2127
FASLG	15	5210.263877
BMP2	15	5337.049932
LDLR	15	2191.70904
HDAC9	15	10403.97449
GRM5	14	3250.915796
CASP8	14	5874.766056
ESR1	14	11585.62988
NFATC2	14	4879.928683
PBK	14	4821.131434
VAV2	14	8716.774939
GRM1	14	3250.915796
FGFR1OP	13	6727.611655
DTL	13	2540.356126
TGFBR1	13	4022.943631
CAMK2D	13	5055.739472
CASP9	12	4919.739038
P2RY6	12	2304
YWHAZ	11	4040.292887
MAP3K7	11	6201.810194
FLT1	11	6634.16899
PPP1CB	11	2005.113067
NCOA3	11	7457.814126
FGFR2	11	3030.464668
ITGA2	10	3529.023629
NOTCH2	10	10947.19567
CENPN	10	1873.156984
FGFR1	10	8007.32537
IRF4	10	5202.998605
PTBP1	10	6730.910039
MEF2C	10	4165.497288
ARHGEF7	9	3147.468396
TIAM1	9	4058.542014
ERBB4	9	4519.484052
ARCN1	9	2088.364196
CASP7	9	5818.243304
PGR	9	4778.496029
SPTBN1	9	10172.1474
DMD	9	2456.15705
FZD3	9	2260.879218
MAPK9	9	2693.578423
DYNC1LI2	9	2495.446083
NR3C1	9	4868.398944
DVL3	9	3925.165711
HDAC8	9	2385.834093
APC	9	4483.983598
TNRC6A	8	2766.776691
RPS6KA1	8	2691.499073
TJP1	8	4534.026739
ACVR1B	8	1933.546424
TGFB2	8	2116.806735
SP100	8	2757.903141
POLR1E	7	6748.802196
ETS1	7	4087.280316
CSNK1A1	7	2157.303406
PSEN1	7	3233.020264
TAF1	7	4214.786588
ERC1	7	3537.432599
CCNE2	7	1750.370853
REL	7	2794.403409
MAPT	7	2044.275299
LIMK1	7	1750.999958
CSF2RA	7	1782.306466
E2F2	7	4585.295895

Twelve topological algorithms were applied and the top 20 genes of each method for the subnetwork of PPI were extracted. These selected genes that appeared at least twice are conserved as hub genes (Table 4).

**Table 4:** Hub genes identified from top 20 of 12 topological algorithms

Rank	Gene	Counts
1	APP	9
2	MAPK8	9
3	PIK3CG	9
4	PIK3R3	9
5	VEGFA	9
6	ITGB1	8
7	PRKACB	8
8	PTK2	8
9	GNG4	6
10	GSK3B	6
11	PRDM10	6
12	WNT5A	6
13	CDKN1A	5
14	RAD51	5
15	SMURF1	5
16	SMURF2	5
17	CCNF	4
18	CDH1	4
19	ESR1	4
20	H2AFV	4
21	NEDD4	4
22	TRIM36	4
23	BUB1	3
24	EHHADH	3
25	FBXL5	3
26	RAP1A	3
27	RAP1B	3
28	RHOT1	3
29	AGFG1	2
30	ASB13	2
31	FASLG	2
32	FLT1	2
33	KBTBD8	2
34	KIAA0319	2
35	KIF11	2
36	KIF23	2
37	KLHL20	2
38	KLHL5	2
39	LDLR	2
40	LRP2	2
41	MKRN1	2
42	PACSIN1	2
43	PARK2	2
44	PCYT1B	2
45	PGR	2
46	RLIM	2
47	RNF213	2
48	SPSB4	2
49	UBE4A	2

#### Pathway enrichment and crosstalk analysis

Pathway enrichment analysis was also performed by the DAVID database. The results indicate that the pathways of “Signaling pathways regulating pluripotency of stem cells”, “Neurotrophin signaling pathway”, “Proteoglycans in cancer”, and “Hippo signaling pathway” are significantly enriched.

For pathway crosstalk analysis, 45 out of 47 pathways that contain more than two genes met the crosstalk analysis criteria and were selected to construct the network (Fig. 6c). The thickness of the edges indicates measurements of the average value of OC and JC. By using MCODE, a major cluster with 33 nodes and 523 edges was identified from the initial network (Fig. 6d).

#### Comprehensive gene-pathway analysis

After mapping the hub genes into the subnetwork of pathways guided by KEGG, a potential gene-pathway network including 33 essential pathways and 16 hub genes were constructed (Fig. 6e). This network shows that “PIK3R3” and “PIK3CG” participate in most of the pathways. Furthermore, “pathways in cancer” and “focal adhesion” and “proteoglycans in cancer” rank as the top three pathways according to the genes that they involved.

To screen the main factors (including genes and pathways) in the gene-pathway network, those nodes with degree > average were selected (Fig. 6f). It was found that 11 genes (PIK3R3, PIK3CG, MAPK8, GSK3B, ITGB1, CDKN1A, PTK2, VEGFA, PRKACB, RAP1B, and RAP1A) with five pathways (pathways in cancer, focal adhesion, proteoglycans in cancer, PI3K-Akt signaling pathway, and Ras signaling pathway) are involved and considered more likely to play a role as influential agents.

#### Identification of key genes and pathways

**Fig. 7:**
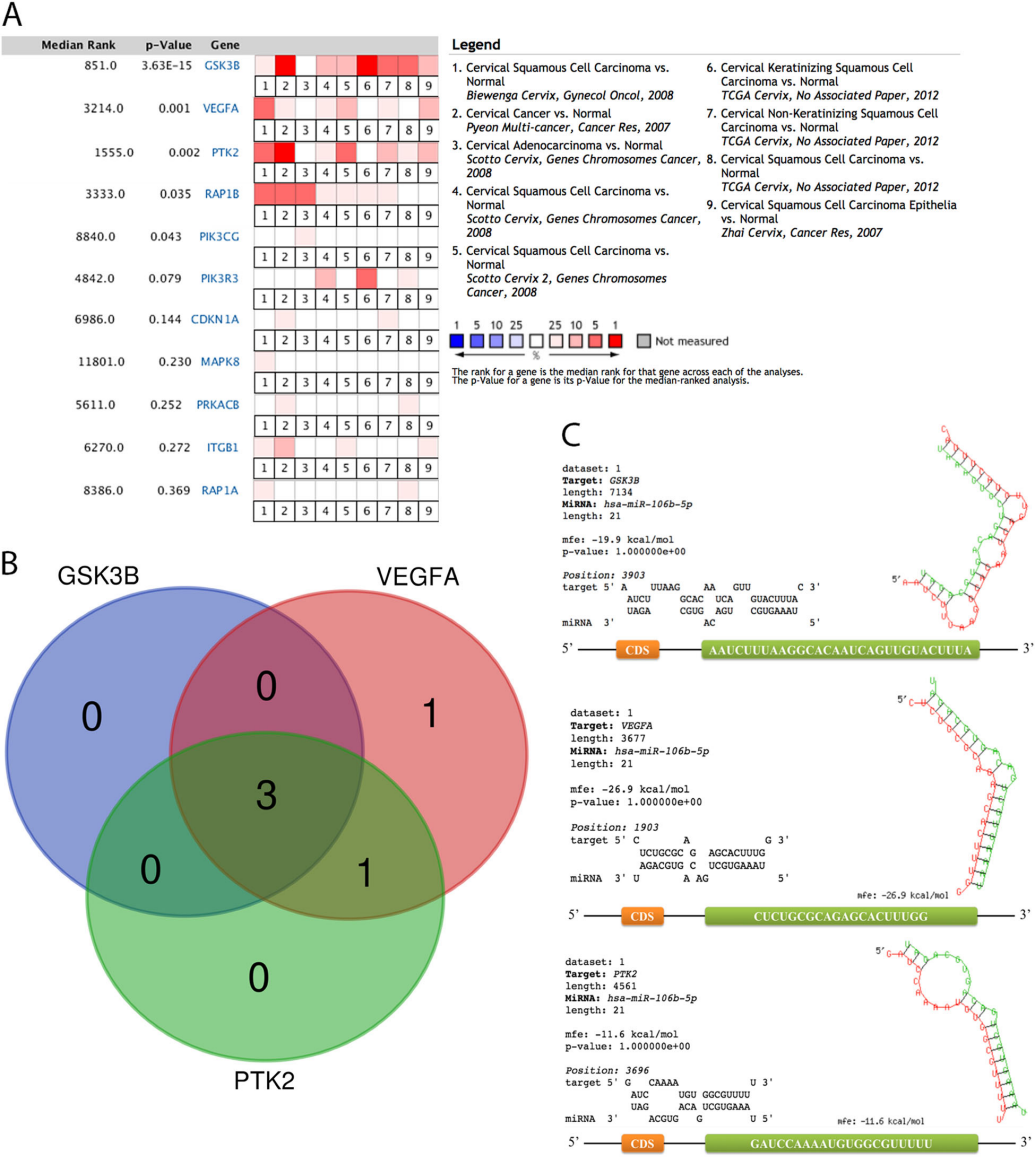
The expression of main genes of cervical cancer across nine studies (**a**), Venn plot for the interaction between key genes and their pathways (**b**), and the binding sequence and location between miR-106b-5p and each critical gene (**c**). Orange square: coding sequence (CDS). Green square: binding site

The expression of the main genes was investigated with meta-analysis through nine studies in Oncomine. The results show that all of the main genes are upregulated in CC, among which five genes (GSK3B, VEGFA, PTK2, RAP1B, and PIK3CG) gain significant differences (Fig. 7a). However, the influence analysis indicates that RAP1B and PIK3CG obtain a discrepant result with the meta-analysis for the altering of significance (Table 5). Hence, only GSK3B, VEGFA, and PTK2 are considered to be key genes.

We merged the pathways that each essential gene participates in order to discover the most important pathways. Three pathways, PI3K-Akt signaling pathway, focal adhesion, and pathways in cancer, which all of the three key genes involved in, were identified (Fig. 7b).

#### Location and characteristic of the binding site

As presented in Fig. 7c, all of the binding sites for miR-106b-5p are located in 3′UTR of GSK3B, VEGFA, and PTK2. Furthermore, when inspecting the sequence of binding sites, adenine (A) and uracil (U) are occupied in most of the sequence.

## Discussion

This study confirms that the expression of miR-106b-5p is significantly upregulated in CC and its expression is highly correlated with the number of metastatic lymph nodes. By using bioinformatics analyses, miR-106b-5p is found to be a key issue in the progression of CC by interacting with three key genes and pathways.

Our results show that miR-106b-5p promotes the progression of CC by targeting GSK3B, VEGFA, and PTK2. GSK3B is dysregulated in a variety of tumor tissues including CC^19–24^, and it participates in the development of CC caused by HPV16 infection by regulating Wnt signaling/β-catenin pathway^23,24^. In addition, GSK3B exerts antiproliferative effects by promoting APC-dependent phosphorylation and thus promotes protease-mediated degradation of β-catenin which is a transcription factor that positively regulates Myc and cyclin D1 expression^21^. When referring to VEGFA, its overexpression in CC can enhance the growth and invasion of tumor cells^25^. More importantly, VEGFA can promote the proliferation and migration of CC cells by activating the PI3K/Akt/mTOR pathway^26^. In addition, the expression and stability of VEGFA have a close relationship with cellular hypoxia^27^ and serum VEGFA level can be used as a biomarker for prognosis evaluation^28^. In terms of PTK2, it is a cytoplasmic protein tyrosine kinase and has an expression in various solid tumors such as ovarian cancer, gastric cancer, and bladder cancer^29^. It is found that PTK2 is associated with the sensitivity of colon cancer cells to DNA damage therapy^30^. However, there are few reports of PTK2 in CC.

The results of pathway analyses reveal that all of the three key genes are involved in the pathway of PI3K-Akt signaling pathway, focal adhesion, and Pathways in cancer. With regard to PI3K-Akt signaling pathway, its imbalance in expression is associated with a variety of tumors, including cervical, endometrial, and non-small cell lung cancers^31–33^. It is activated during the G1/S transition of the cell cycle and regulates several key cell cycle regulators^34^. Tumors associated with HPV infection are closely related to the PI3K/Akt pathway. Activation of this pathway contributes to genetic instability, dysregulation, apoptosis resistance, and altered metabolic properties ultimately leading to the malignant transformation of infected cells. Concerning focal adhesion, this pathway is involved in the invasion and metastasis of many kinds of tumors and is related to the medicine resistance of certain tumors^35,36^. The decreased expression of focal adhesion kinase (FAK), a key gene involved in the pathway, can inhibit the invasion and migration of CC cells^37^. Another study reports that targeted FAK therapy makes pancreatic cancer cells more sensitive to immunotherapy^38^.

It has been recognized that miRNA negatively regulates gene expression by guiding the RNA-induced silencing complex (RISC) to silence its target mRNA through degradation or translation repression^39^. However, it is interesting to observe that both the expression of miR-106b-5p and key genes are upregulated in CC with the binding sites located in 3′UTR of mRNAs. According to previous studies^40–44^, the mechanisms of miRNA-mediated gene upregulation include (1) the presence of cellular state (G0 or G0-like state) and/or specific factors (AGO1, AGO2, GW182, and FXR1); (2) miRNA directly binds to 5′UTR of RNA and increases its association with 40S and polysome formation or enhances their translation by alleviating their TOP-mediated translational repression during amino acid starvation, respectively; (3) miRNA prevents tristetraprolin (TTP) binding to the AU-rich element (ARE) sites of mRNA and inhibits its degradation by ARE-mediated mRNA decay (AMD) pathway; (4) derepression from miRNA-mediated downregulation in response to cell stresses by HuR (an RNA binding protein that interacts with ARE in 3′UTR of the mRNA). As none of the binding sites are located in 5′UTR in our results, and cells are considered active in cancer tissue, the mechanisms of upregulation for cellular state or binding site in 5′UTR may be less possible.

AREs are found in the 3′UTR of mRNAs that code for proto-oncogenes, nuclear transcription factors, and cytokines^45^. They can be classified into three types: (1) having dispersed AUUUA motifs within or near U-rich regions; (2) having overlapping AUUUA motifs within or near U-rich regions; (3) a much less well-defined class having a U-rich region but no AUUUA repeats^46^. Our results reveal that A and U are occupied in most of the binding sequence. To our knowledge, it has been verified that TTP has interactions with GSK3B^47^ and VEGFA^48^ whereas there are no reports on PTK2. It is possible that miR-106b-5p prevents TTP from binding to the mRNAs in 3′UTR and therefore regulate their expression, but the specific mechanism needs to be further investigated. In addition, it has been confirmed that HuR can bind to VEGF^49^ and reverse the repression effect by miR-200b^50^. This also could be a contributing factor to the upregulation of VEGFA and be adopted for the other key genes.

However, several limitations exist in the present study: (1) Significant heterogeneity can be observed in the meta-analysis. This high heterogeneity may result from population, race, stage, and the type of CC (squamous or adenocarcinoma). Hence, more data from large-scale clinical trials are needed to evaluate the source of the heterogeneity. (2) Parts of genes are removed due to the selection criteria. However, it is possible that these genes might also impact the progression of CC and they also need to be analyzed. (3) As this study is an in silico research, a further experiment is needed for validation. (4) Upregulations of both miR-106-5p and key genes are identified. Despite the fact that several possibilities are analyzed, the specific mechanisms still need to be studied and verified further.

## Conclusions

In summary, significant upregulation of miR-106b-5p in CC is confirmed by meta-analysis with the data from TCGA and GEO, and the expression of miR-106b-5p is significantly correlated with the number of metastatic lymph nodes. Furthermore, miR-106b-5p promotes the progression of CC by targeting three key genes (GSK3B, VEGFA, PTK2) through three crucial pathways (PI3K-Akt signaling pathway, focal adhesion, and pathways in cancer). miR-106b-5p might upregulate the key genes by preventing TTP from binding to the mRNAs in 3’UTR with/without the effect of derepression of HuR. However, the specific mechanism needs to be further investigated.

## Materials and methods

**Fig. 8:**
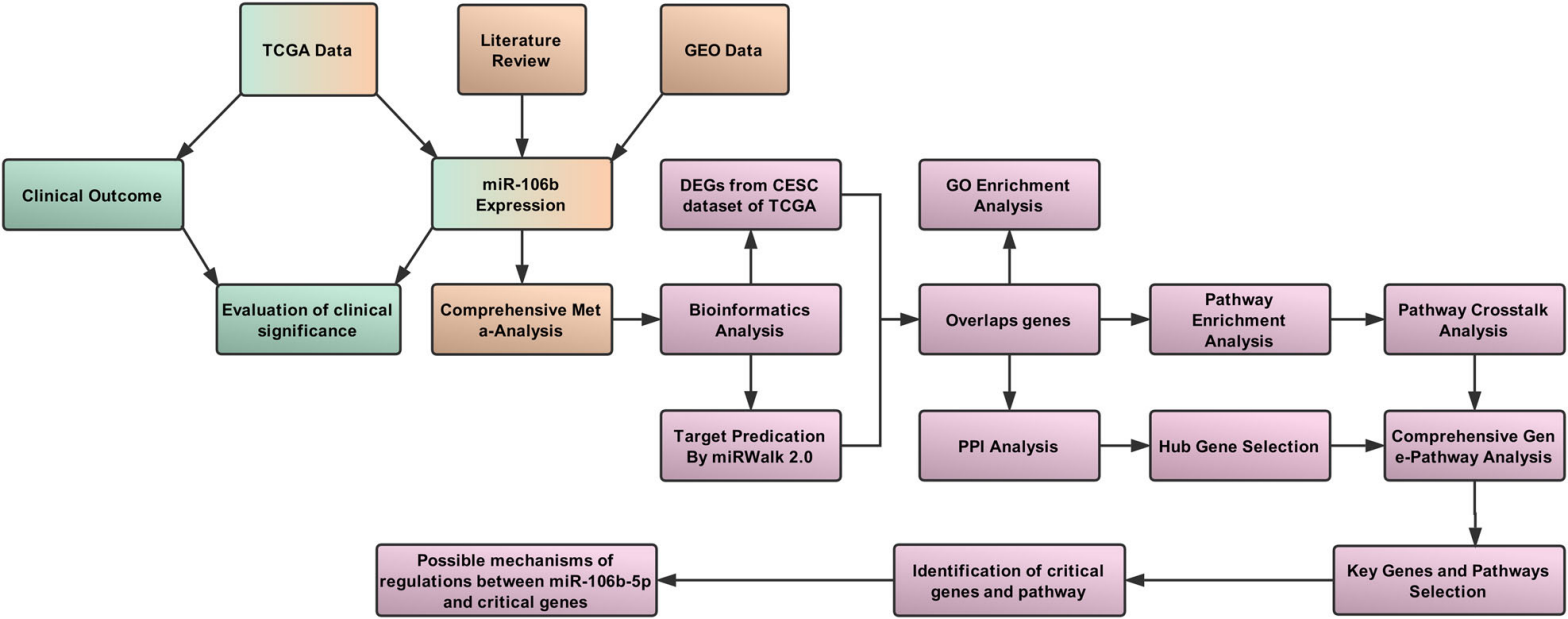
Work flow of the clinical significance evaluation and comprehensive analysis for miR-106b-5p in cervical cancer. The modules of clinical value evaluation (green), meta-analysis (brown), and bioinformatics analyses (pink) are included

The workflow of this study is presented in Fig. 8 and this study is performed according to guidelines of MIAME^51^ and Meta-Analysis of Gene Expression Microarray Datasets^52^. Firstly, the clinical significance of miR-106b-5p in CC is assessed according to the CESC data from TCGA. Secondly, the expression data of miR-106b-5p from TCGA, GEO, and literature are synthesized by meta-analysis. Thirdly, DEGs from TCGA are screened and 12 databases are used to predict target genes of miR-106b-5p. The overlap genes between DEGs from TCGA and predicted target genes are explored by bioinformatic analyses.

### Correlations between the expression of miR-106b-5p and clinical outcomes

To identify the clinical significance of miR-106b-5p in CC, we adopt LinkedOmics^53^ which is constructed based on TCGA with 309 cancer samples and three normal samples as controls in CESC dataset to explore the correlations between the expression of miR-106b-5p and the clinical outcomes, including the number of metastatic lymph nodes, tumor purity, race, pathology M/N/T stage, years to birth, histological type, ethnicity, radiation therapy, and overall survival.

### Meta-analysis of miR-106b-5p expression based on TCGA/GEO and literature

A systematic searching in GEO for the miR-106b-5p expression between CC and healthy tissue was performed on 30 March 2018 with the terms and eligibility criteria that are presented in Tables 5 and 6. For adjusting the data to the normal distribution to reduce variation, Log2 scale transformation was applied to all of the expression data. The meta-analysis was conducted by R Version 3.4.1^54^ and meta package^55^. We pooled SMD using the Mantel–Haenszel formula (fixed-effect model) or the DerSimonian–Laird formula (random effects model) and assessed the heterogeneity using *I*^2^ statistics. Random-effect models (*P* < 0.05 or *I*^2^ > 50%) are used when the heterogeneity is considered significant. Otherwise a fixed-effects model would be used.

**Table 5:** Influence analysis of 5 key genes

Omitting study	P_GSK3B_	P_VEGFA_	P_PTK2_	P_RAP1B_	P_PIK3CG_
Omitting Study 1	2.16*10^-6^	0.023	9.22*10^-4^	0.067	0.165
Omitting Study 2	1.88*10^-4^	0.023	9.22*10^-4^	0.067	0.489
Omitting Study 3	2.16*10^-6^	7.55*10^-4^	9.51*10^-4^	0.067	0.367
Omitting Study 4	2.16*10^-6^	0.022	9.51*10^-4^	0.023	0.165
Omitting Study 5	1.88*10^-4^	0.023	9.22*10^-4^	0.067	0.367
Omitting Study 6	1.88*10^-4^	7.55*10^-4^	9.51*10^-4^	0.023	0.165
Omitting Study 7	1.88*10^-4^	7.55*10^-4^	2.97*10^-5^	0.055	0.367
Omitting Study 8	1.90*10^-4^	7.55*10^-4^	9.51*10^-4^	0.023	0.165
Omitting Study 9	2.16*10^-6^	0.023	9.22*10^-4^	0.023	0.367

Study 1: Cervical Squamous Cell Carcinoma vs. Normal .Biewenga Cervix, Gynecol Oncol, 2008

Study 2: Cervical Cancer vs. Normal. Pyeon Multi-cancer, Cancer Res, 2007

Study 3: Cervical Adenocarcinoma vs. Normal. Scotto Cervix, Genes Chromosomes Cancer, 2008

Study 4: Cervical Squamous Cell Carcinoma vs. Normal. Scotto Cervix, Genes Chromosomes Cancer, 2008

Study 5: Cervical Squamous Cell Carcinoma vs. Normal. Scotto Cervix 2, Genes Chromosomes Cancer, 2008

Study 6: Cervical Keratinizing Squamous Cell Carcinoma vs. Normal. TCGA Cervix, No Associated Paper, 2012

Study 7: Cervical Non-Keratinizing Squamous Cell Carcinoma vs. Normal. TCGA Cervix, No Associated Paper, 2012

Study 8: Cervical Squamous Cell Carcinoma vs. Normal. TCGA Cervix, No Associated Paper, 2012

Study 9: Cervical Squamous Cell Carcinoma Epithelia vs. Normal. Zhai Cervix, Cancer Res, 2007.

P_Gene_: The p-Value for a gene and is the p-Value for the median-ranked analysis

**Table 6:** Searching terms used in GEO and literature review

(1) Microarray searching	
#1	microRNA OR miRNA OR micro RNA noncoding RNA OR ncRNA OR small RNA
#2	Cervical OR cervix
#3	Cancer OR carcinoma OR tumor OR neoplasia OR neoplasm OR malignant OR malignancy
#4	#1 AND #2 AND #3
(2) Literature search	
#1	microRNA OR miRNA OR micro RNA noncoding RNA OR ncRNA OR small RNA
#2	106b OR 106b-5p
#3	Cervical OR cervix
#4	Cancer OR carcinoma OR tumor OR neoplasia OR neoplasm OR malignant OR malignancy
#5	#1 AND #2 AND #3 AND #4

Funnel plot with Egger’s test^56^ was utilized to evaluate the publication bias. *P* < 0.1 was considered to be significant asymmetry for the funnel plot.

To detect the robustness of the pool results, sensitivity analysis was performed by alternating analysis model. In addition, to further evaluate the impact of individual studies on the overall effect estimates, influence analysis was performed and the combined estimates were recalculated by omitting one study at a time.

Furthermore, a full-scale search of miR-106b-5p expression in eight electronic databases (PubMed, Chinese VIP, CNKI, Wanfang Database, Embase, Web of Science, Science Direct, and Wiley Online Library) accompanied with manual searching by screening the references cited in the acquired articles was conducted on 30 March 2018. The searching terms and eligible criteria are also shown in Tables 5 and 6. The data of the publications will be extracted and analyzed, including the author, year of publication, country, number of cancer and control samples, regulations, and testing methods.

### Screening of candidate genes for miR-106b-5p

The CESC data were analyzed by the limma package^57^ to identify DEGs. We determined the significance of the difference in gene expression as Log2 |fold change (FC) | > 1 and False Discovery Rate (FDR) < 0.05. Furthermore, the miR-106b-5p targeted genes are predicted by 12 databases (Microt4, miRWalk, mir-bridge, miRanda, miRDB, miRMap, Pictar2, PITA, miRNAMap, RNAhybrid, RNA22, and Targetscan) in miRWALK version 2.0^58^. To increase prediction accuracy, the genes that are overlapping in at least five databases were selected. Finally, the overlap genes between DEGs and predicted genes were analyzed by upsetR^59^ and Venn Plot.

### GO and pathway enrichment analysis

GO and pathway enrichment analyses were performed by DAVID^60^ on the overlapping genes. The significantly enriched biological items for CeC, BP, and molecular functions (MF) were identified as *P* < 0.01.

### Protein-protein interaction network analysis

The STRING database^61^ was used to construct an interaction network between the overlapped genes. To obtain precise results, those nodes in the network would be removed: (a) interaction score < 0.7; (b) not connected to the major network; (3) the value of degree and betweenness is less than average.

CytoHubba^62^ was used to explore the important nodes by 12 topological algorithms, including Degree, Edge Percolated Component (EPC), Maximum Neighborhood Component (MNC), Density of Maximum Neighborhood Component (DMNC), Maximal Clique Centrality (MCC), and centralities based on shortest paths, such as Bottleneck (BN), EcCentricity, Closeness, Radiality, Betweenness, ClusteringCoefficient, and Stress. The top 20 genes in each topological algorithm were extracted and the duplication of each gene was also calculated. The genes with less than 2 in repetitiveness are excluded for ensuring the genes are closely linked to CC and the rest are considered as hub genes.

### Pathway enrichment and crosstalk analysis

For pathway enrichment, the predicted targets were mapped using the Kyoto Gene and Genome Encyclopedia (KEGG) database by online analysis of DAVID. Significant pathways were considered as *P* < 0.05.

The obtained pathways were recruited for further crosstalk analysis to explore their interactions. The method is based on the assumption that if two pathways share a proportion of genes, they are considered crosstalk^63^. As described in the previous study^64^, in order to better measure the overlap between any two pathways, JC (Jaccard coefficient) = | (A ∩ B) / (A ∪ B) | and OC (overlapping coefficient) = (|A ∩ B|) / (min(| A |, | B |)) were adopted, where A and B are the genes contained in the two pathways. As pathways with too few genes may have insufficient biological information, we excluded pathways containing fewer than three genes. Likewise, pairs of pathways with less than two overlapping genes were also removed. After obtaining the network of pathway crosstalk, the plug-in app Molecular Complex Detection (MCODE) in Cytoscape was used to screen the hub subnetwork which had the score >4.

### Comprehensive gene-pathway analysis

The hub genes are mapped into the subnetwork of crosstalk to further explore the mechanism by KEGG. To screen the main genes and pathways, the nodes with degree > average are collected for constituting a subnetwork.

### Identification of key genes and pathways

To further identify the key genes, we evaluated the expression of main genes between CC and healthy samples with meta-analysis in Oncomine^65^. *P* < 0.05 is considered as a significant difference. Moreover, influence analysis was also conducted to access the pool estimates. The pathways that all of the key genes participate in were determined as crucial pathways.

### Possible mechanisms of regulations between miR-106b-5p and key genes

The regulation between miR-106b-5p and the key genes is recognized by their expression. To explore the possible mechanisms of the regulation, we collected the sequence of miR-106b-5p and the three key genes from miRbase^66^ and NCBI–nucleotide to predict their binding site by RNAhybrid^67^ with the criteria that mRNA has perfect nucleotide pairing between the second and eighth positions of the 5′ end of miRNA sequences. Furthermore, the character of the binding sequence was also investigated.

## Electronic supplementary material

Author contribution form
