# Supplementary material for: The role of miR-106p-5p in cervical cancer: from expression to molecular mechanism
Source: Cell Death Discov. 2018 Sep 20;4:94. doi: 10.1038/s41420-018-0096-8 (PMC6148547; doi:10.1038/s41420-018-0096-8)
Supplement: Supplementary file 1 — Author contribution form [file 41420_2018_96_MOESM1_ESM.pdf]

DECLARATION OF CONTRIBUTIONS TO ARTICLE

ADMC

Manuscript Number: CDDIS-18-1695

Journal Name: *Cell Death & Disease* (the 'Journal')

Proposed Title of the Contribution:

The role of miR-106p-5p in cervical cancer: from expression to molecular mechanism (the 'Contribution)

Author(s):

Yuexiong Yi, Yanyan Liu, Wanrong Wu, Kejia Wu, Wei Zhang (the 'Authors')

For all *CDDis* articles, each person named as an author in the published version must be able to show he or she has contributed substantially to the article.

Authorship credit should be based on 1) substantial contributions to conception and design, acquisition of data, or analysis and interpretation of data; 2) drafting the article or revising it critically for important intellectual content; and 3) final approval of the version to be published. Authors should meet conditions 1, 2 and 3.

Any person who cannot be shown to have made a substantial contribution to the article cannot be listed as an author in the final version. The name of any person who is deemed to have made a minor contribution can, however, appear in the Acknowledgments section of the article.

Please complete the table below to indicate the contributions of all named authors to the manuscript.

Author Full Name:	Specification of Contribution to the Manuscript:
Yuexiong Yi	Design the study and write the paper; Perform he statistical analysis
Yanyan Liu	Collect the data and perform the bioinformatics analysis
Wanrong Wu	Collect the data and perform the bioinformatics analysis
Kejia Wu	Collect the data and perform the bioinformatics analysis; Perform he statistical analysis
Wei Zhang	Design the study and write the paper

Please complete the table below to indicate the contributions of all named authors to the figures.

Figure 1:

In Figure 1, Yuexiong Yi and Wei Zhang design the whole process and Yanyan Liu construct the plot.

Figure 2:

In Figure 2, Yanyan Liu collect the data and Wanrong Wu generate the plot by using R

Figure 3:

In Figure 3, Yanyan Liu, Wanrong Wu and Kejia Wu screen the microarray and literature and Kejia Wu construct the work flow

Figure 4:

In Figure 4, Yanyan Liu, Wanrong Wu and Kejia Wu collect the data from TCGA, GEO and literature, and Yuexiong Yi construct the plot by using R

Figure 5:

In Figure 5, Wanrong Wu and Kejia Wu collecting the data from miRWalk and TCGA, and Wanrong Wu generate the plot.

Figure 6:

In Figure 6, Kejia Wu collect the data from DAVID and Yuexiong Yi construct the plot. In Figure 7, Yanyan Liu and Wanrong Wu collect the data and Yuexiong Yi with Kejia Wu perform the networking construction and statistical analysis. In Figure 8, Yanyan Liu and Wanrong Wu collect the data from oncomine, miRBase and NCBI, and Yuexiong Yi generate the plot

Signed for and on behalf of the Author(s):	Print Name:	Date:
Wei Zhang	Wei Zhang	2018.06.08
